# Multifractal Functional Connectivity Analysis of Electroencephalogram Reveals Reorganization of Brain Networks in a Visual Pattern Recognition Paradigm

**DOI:** 10.3389/fnhum.2021.740225

**Published:** 2021-10-18

**Authors:** Orestis Stylianou, Frigyes Samuel Racz, Keumbi Kim, Zalan Kaposzta, Akos Czoch, Andriy Yabluchanskiy, Andras Eke, Peter Mukli

**Affiliations:** ^1^Department of Physiology, Semmelweis University, Budapest, Hungary; ^2^Institute of Translational Medicine, Semmelweis University, Budapest, Hungary; ^3^Vascular Cognitive Impairment and Neurodegeneration Program, Department of Biochemistry and Molecular Biology, Oklahoma Center for Geroscience and Healthy Brain Aging, The University of Oklahoma Health Sciences Center, Oklahoma City, OK, United States; ^4^The Peggy and Charles Stephenson Cancer Center, The University of Oklahoma Health Sciences Center, Oklahoma City, OK, United States; ^5^Department of Health Promotion Sciences, College of Public Health, The University of Oklahoma Health Sciences Center, Oklahoma City, OK, United States; ^6^Department of Radiology and Biomedical Imaging, Yale University School of Medicine, New Haven, CT, United States

**Keywords:** multifractal, functional connectivity, brain networks, electroencephalography, visual pattern recognition

## Abstract

The human brain consists of anatomically distant neuronal assemblies that are interconnected via a myriad of synapses. This anatomical network provides the neurophysiological wiring framework for functional connectivity (FC), which is essential for higher-order brain functions. While several studies have explored the scale-specific FC, the scale-free (i.e., multifractal) aspect of brain connectivity remains largely neglected. Here we examined the brain reorganization during a visual pattern recognition paradigm, using bivariate focus-based multifractal (BFMF) analysis. For this study, 58 young, healthy volunteers were recruited. Before the task, 3-3 min of resting EEG was recorded in eyes-closed (EC) and eyes-open (EO) states, respectively. The subsequent part of the measurement protocol consisted of 30 visual pattern recognition trials of 3 difficulty levels graded as Easy, Medium, and Hard. Multifractal FC was estimated with BFMF analysis of preprocessed EEG signals yielding two generalized Hurst exponent-based multifractal connectivity endpoint parameters, *H*(2) and Δ*H*_15_; with the former indicating the long-term cross-correlation between two brain regions, while the latter captures the degree of multifractality of their functional coupling. Accordingly, *H*(2) and Δ*H*_15_ networks were constructed for every participant and state, and they were characterized by their weighted local and global node degrees. Then, we investigated the between- and within-state variability of multifractal FC, as well as the relationship between global node degree and task performance captured in average success rate and reaction time. Multifractal FC increased when visual pattern recognition was administered with no differences regarding difficulty level. The observed regional heterogeneity was greater for Δ*H*_15_ networks compared to *H*(2) networks. These results show that reorganization of scale-free coupled dynamics takes place during visual pattern recognition independent of difficulty level. Additionally, the observed regional variability illustrates that multifractal FC is region-specific both during rest and task. Our findings indicate that investigating multifractal FC under various conditions – such as mental workload in healthy and potentially in diseased populations – is a promising direction for future research.

## Introduction

The human brain is a complex system encompassing spatially distinct neuronal populations interconnected via an intricate axonal grid. Functional brain networks emerge within this anatomical circuitry, which provides the neurophysiological basis for higher-order brain functions (Van Hoesen, 1993). For instance, visual pattern recognition requires coordinated interactions among disparate brain regions such as the visual cortex, where primary processing, and the association areas in the parietal and frontal cortices, where high-level cognitive evaluation takes place (Van Hoesen, 1993; Kandel et al., 2012). Based on the hypothesis that regions that exhibit statistically similar dynamics are functionally coupled, functional neuroimaging methods allowed the reconstruction of functional connectivity (FC) in the brain under cognitive (Friston et al., 1993) and motor (Biswal et al., 1995) tasks. A paradigm shift regarding resting-state studies occurred after discovering that even in the absence of external stimuli the brain is organized in resting-state networks (RSNs) (Raichle et al., 2001). This resting-state neural architecture is altered during task through a series of activations and deactivations of brain regions (Fox et al., 2005). Accordingly, we believe that studying the brain under mental workload could reveal valuable information.

Due to its high spatial resolution, functional magnetic resonance imaging (fMRI) has been commonly favored as the gold standard imaging technique for detecting task-related changes of FC (Fox et al., 2005; Krienen et al., 2014; Di et al., 2015; Elton and Gao, 2015; Kaufmann et al., 2017). Nevertheless, the low sampling frequency and the physical constraints of the fMRI systems present themselves as limitations when more elaborate experimental paradigms are designed. Albeit at the cost of a lower anatomical resolution, these limitations can be overcome using electroencephalography (EEG) owing to its high sampling rate and easy-to-use instrumentation. This led to numerous task-related EEG studies, ranging from traditional tasks like n-back (Hou et al., 2018; Kaposzta et al., 2021) and face perception (Yang et al., 2015) to more complex designs like urban navigation (Skroumpelou et al., 2015). By using a visual pattern recognition paradigm, Racz et al. demonstrated an increase in scale-specific FC during task (Racz et al., 2017); though, in that study the scale-free aspect of the connections was not taken into consideration.

Various statistical approaches have been applied and/or developed for characterizing the linear and nonlinear aspects of the coupled neural activities (Bastos and Schoffelen, 2016). A common limitation of these methods is that they capture interdependence on a single scale, despite the fact that the scale-free (or fractal) nature of the connections has already been demonstrated in various modalities such as EEG (Wang and Zhao, 2012; Stylianou et al., 2021), fMRI (Ciuciu et al., 2014) and magnetoencephalography (Achard et al., 2008). The univariate scale-free behavior of neural dynamics has already been shown both regionally (Popivanov et al., 2006) and globally (Stam and de Bruin, 2004). While estimating FC at a given time scale reflect the coupling between oscillatory (narrowband) components at specific cross-spectrum frequencies, our current approach assumes a significant scale-free (broadband) component of the cross-spectrum; a signature of statistical dependency spanning a broad range of frequencies (scales). Moreover, the true multifractal nature of coupled dynamics was recently validated in resting-state EEG (Stylianou et al., 2021). Scale-free FC estimators allow for capturing how the long-term memory and multifractality of the coupled dynamics are spatially distributed across brain networks; topological aspects that otherwise would remain obscured. Visual pattern recognition requires sustained interaction between brain regions involved in the processing of the visual information, which can be captured as increased cross-correlations (long-term memory) in the functional connections. Furthermore, cognitive stimulation implies a shift in FC that is typically governed by complex nonlinear dynamics (Rabinovich and Muezzinoglu, 2010; Werner, 2010), which might alter the multifractal profile of FC. To the best of our knowledge, this is the first study investigating the task-related network reorganization using multifractal connectivity analysis.

In the current study, we examined the task-related reorganization of FC by applying a bivariate, focus-based adaptation of multifractal analysis on EEG records. The task of choice was a complex pattern recognition paradigm, which has previously shown its utility in increasing FC in the prefrontal cortex (Racz et al., 2017). Our primary objectives were: (i) to test the hypothesis that shifts in scale-free coupled dynamics would occur during the transition from rest to task; and (ii) to examine the localization of multifractal FC within each mental state. Our secondary aim was to assess the relationship between cognitive performance and brain network measures reconstructed from scale-free FC estimators.

## Materials and Methods

### Participants

Fifty-eight young, healthy volunteers (24.2 ± 3.4 years of age, 28 females, 9 left-handed) with no history of psychiatric/neurological illness were recruited for the study. Participants were instructed to have a good night’s sleep before the day of the experiment. All subjects provided written informed consent prior to the measurement. The study was designed and carried out in accordance with the Declaration of Helsinki and was approved by the Regional and Institutional Committee of Science and Research Ethics of Semmelweis University (approval number: 2020/6).

### Measurement Protocol

**FIGURE 1:**
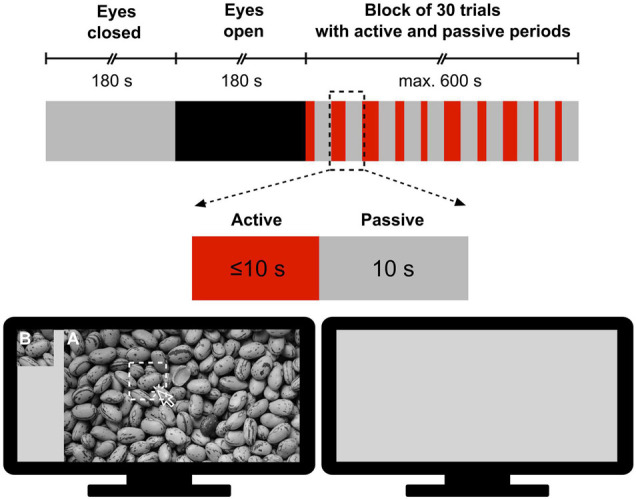
Measurement protocol for obtaining electroencephalography records during resting states and subsequent visual pattern recognition. First, resting-state recordings were made in 180 s periods with eyes closed and eyes open, respectively. Then, the subject performed a pattern recognition task in a block of 30 trials, each consisting of a 10 s or less of active period and a 10 s passive period. In the active period, participants were presented a large-size image **(A)** and its cropped sub-region **(B)** and were required to click on **(A)** at the position of **(B)** if found *(The picture of Figure 1 was taken from https://alphacoders.com/users/profile/97828)*.

All measurements took place in the Department of Physiology at Semmelweis University in a quiet room under subdued ambient illumination. During the measurement, participants were seated comfortably in a chair in front of a computer monitor at an approximate distance of 0.8 m from the screen and were instructed to refrain from moving and facial expressions as much as possible. The measurement protocol was designed and implemented in MATLAB (version 2012, Mathworks, Natick, MA, United States) according to a pattern recognition paradigm modified after Racz et al. (2017). The session started with a 3-min eyes-closed (EC) period serving as a baseline, followed by a 3-min eyes-open (EO) resting-state period, as a control for the task state. Then, participants were engaged in a visual pattern recognition paradigm consisting of a block of 30 trials with active and passive periods (Figure 1). Specifically, in the active period of a trial, the subject was allowed a maximum of 10 s to identify a sub-region of a grayscale image by clicking on its assumed location; at that point, the active period was terminated. The active period was immediately followed by a passive (task-free) period, during which a gray background was displayed for 10 s. In this stimulation paradigm, a pool of 6 different grayscale images was permutated. Each of them was shown 5 times in total – with a different sub-region to be identified in each case – thus yielding a total of 6 × 5 = 30 trials. To investigate the impact of difficulty level, images were sorted into Easy, Medium and Hard categories with 2 images in each. Their classification was based on their complexity, defined as the file size ratio of compressed/uncompressed images [cf. Equation 1 in Yu and Winkler (2013)]. The order of the 30 trials was randomized with a different permutation sequence for each participant. The following metrics characterized the performance during pattern recognition: (i) reaction time, defined as the time between the beginning of the image presentation and response (left mouse click on the image) and (ii) success, defined as 1 if the participant correctly identified the sub-region’s location and 0 otherwise. When the subject did not respond, the trial was considered a failure (success = 0) and the reaction time was set to 10 s.

### Data Acquisition

EEG signals were recorded by a wireless Emotive Epoc+ device and its corresponding EmotivPRO software (Emotiv Systems Inc., San Francisco, CA, United States). After ensuring low electrical impedance (<20 kΩ), EEG signals from 14 brain regions (10--20 standard montage locations: AF3, AF4, F3, F4, F7, F8, FC5, FC6, T7, T8, P7, P8, O1, and O2) were recorded, at a 128 Hz sampling rate^1^. CMS and DRL electrodes at left and right mastoid processes were used as reference.

### Preprocessing

The EEG device applied built-in band-pass (0.2–45 Hz, digital 5th order Sinc) and notch (50 and 60 Hz) filters to the raw data. To maximize the artifact-detection capacity of independent component analysis (ICA), first we performed wavelet-enhanced ICA (wICA) (Rong-Yi and Zhong, 2005; Gabard-Durnam et al., 2018). The purpose of wICA was to exclude wavelet components with coefficients higher than a certain threshold, resulting in the removal of high amplitude spikes. Subsequently, we manually excluded non-brain components, as ICA isolated them. wICA was performed in an automated manner, while the EEGLAB toolbox (Delorme and Makeig, 2004) was used for manual ICA.

### Estimation of Multifractal Functional Connectivity

**FIGURE 2:**
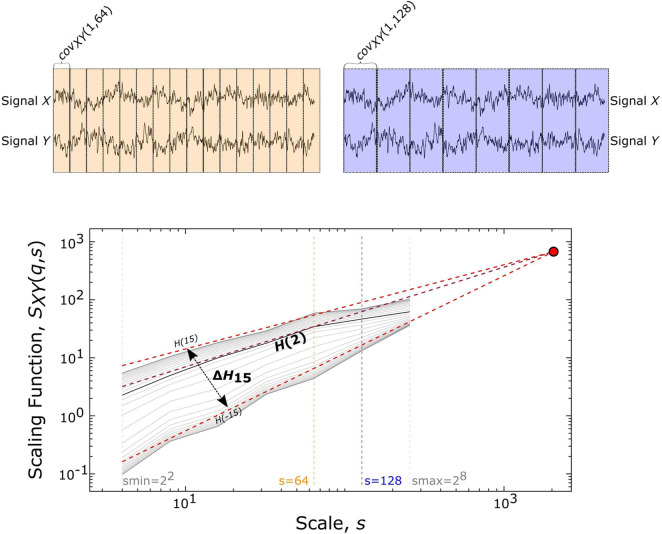
Multifractal time series analysis and its endpoint parameters. On the upper panels, a representative pair of 2048 datapoint-long EEG segments (from Subject01) is displayed along with the windowing scheme for a smaller (*s* = 64, shown in yellow) and larger (*s* = 128, shown in purple) scale, which illustrates the calculation of covariance scaling function [*S*_*XY*_(*q*,*s*) displayed in the lower panel] according to Eq. 1. The *Focus* (red disk) is used as a reference point when simultaneously fitting linear models in the log-log transform of the *S*_*XY*_(*q*,*s*) vs *s*, the essential step of BFMF. The slope of each linear regression line represents the generalized Hurst exponent [*H*(*q*)] (shown for *q* = –15, +2, +15). *H*(2) describes the long-term cross-correlation between the signals *X* and *Y*, while the degree of multifractality (Δ*H*_15_) is captured in the difference between *H*(*q*) values at the extreme [i.e., minimal (–15) and maximal (15)] statistical moments.

The scale-free coupled dynamics were estimated with bivariate focus-based multifractal analysis (BFMF), introduced by Mukli and colleagues (Mukli et al., 2018). The applicability of BFMF for multifractal FC estimation was demonstrated previously (Stylianou et al., 2021). Here we only provide a summary of the method, while further details are found in the references mentioned above. The scaling function *S*_*XY*_ (Figure 2) of two EEG time series (*X* and *Y*) of length *L* datapoints can be calculated as:


(1) $$S_{X\,Y}\,\left(q,s\right)={\left(\frac{1}{N_{s}}\,\sum_{v=1}^{N_{s}}{\left|c\,o\,v_{X\,Y}\,\left(v,s\right)\right|}^{q}\right)}^{1/q},$$

where *N*_*s*_ denotes the number of bridge-detrended, non-overlapping windows of size *s* (*s* = 2*^*n*^* with *n* being integers ranging from 2 to 8) indexed by *v.* The statistical moment order (*q*) ranges from −15 to 15 with increments of 1 and the window-wise covariance between simultaneous *s*-size segments of *X* and *Y* is denoted by *cov*_*XY*_(*v*, *s*). When *q* = 0, the scaling function takes the form:


(2) $$S_{X\,Y}\,\left(0,s\right)=e^{\left[\frac{1}{2\,N_{s}}\,\sum_{v=1}^{N\,s}\ln\left(\left|c\,o\,v_{X\,Y}\,\left(v,s\right)\right|\right)\right]}$$

In the special case when the whole segment is used for obtaining the scaling function [*S*_*XY*_(*q*,*L*)], the sum in Eq. 1 becomes independent of *q* and thus, the scaling function values of all moments converge to a so-called *Focus*. This *Focus* serves as a reference point when regressing for the log[*S*_*XY*_(*q*,*s*)] vs log[*s*] relationship for every *q* simultaneously. In contrast with the standard approach where separate *q*-wise assessments of the power-law relationship are applied, fitting all statistical moments simultaneously results in a more robust analysis (Mukli et al., 2015). This is achieved by enforcing the monotonous decay of regression slopes, which represent the generalized, *q*-dependent bivariate Hurst-exponent function *H*(*q*). The special case of *H*(2) depicts the global long-term cross-correlation in the coupled dynamics underlying the functional connection. If this bivariate *H*(2) is greater than 0.5, then there is functional coupling exhibiting long-term memory. *H*(2) = 0.5 indicates uncorrelated, uncoupled dynamics, while *H*(2) < 0.5 demonstrates anticorrelated coupling (Eke et al., 2002; Kristoufek, 2014). Δ*H*_15_, calculated as *H*(-15)-*H*(15), captures the degree of multifractality, an indicator of the *q*-wise dependence of the scaling function on large and small covariances. The whole segment of each trial (active section + 10 s of passive period) was analyzed with BFMF. As for the resting-state conditions, 9 non-overlapping segments of 20 s for each of the EC and EO states were analyzed. To remove the effect of different time lengths due to various response times, we also performed analyses adjusted to the length of time series (see Supplementary Material).

### Assessing Multifractality

A series of statistical tests evaluated the true scale-free nature of the connections. In short, the purpose of these tests was to: (i) validate the power-law relationship of the connection both in the frequency and time domains (spectral slope and detrended cross-correlation coefficient tests, respectively), (ii) distinguish true from spurious multifractality (phase randomization and shuffling tests), and (iii) determine if the emerging coupling between the two processes is genuine or only reflects a mere autocorrelation within each EEG signal (bivariate-univariate Hurst comparison). This series of tests reveal the qualitative nature of bivariate multifractality, which is assessed independently from its quantitative changes in this study. The complete account of the testing procedure followed in this study was reported elsewhere (Stylianou et al., 2021). We expanded the test yielding a distinction between extrinsic and intrinsic multifractality referred to as bivariate-univariate Hurst comparison. In our previous paper, only the inequality between the bivariate Hurst exponent and the mean of the univariate Hurst exponents comprising the connection was tested (Stylianou et al., 2021). In the present study, we considered a bivariate-univariate Hurst comparison test successful only when the bivariate *H*(2) was lower than the mean of its univariate *H*(2). This choice was made based on the fact that bivariate *H*(2) can exceed the mean of univariate *H*(2) only due to the finite length or non-normal distribution of the time series (Kristoufek, 2015a, 2016).

### Brain Network Construction

We then proceeded with reconstructing functional networks and analyzing their architecture. For each subject, we isolated 48 different EEG segments (9 EC, 9 EO, 10 Easy, 10 Medium and 10 Hard). For each connection, the *H*(2) and Δ*H*_15_ values obtained in the 5 different states were averaged, resulting in 5 different values per subject. Altogether, 5-5 (i.e., fully connected) networks (EC, EO, Easy, Medium, Hard) were reconstructed for every subject, based on either their *H*(2) or Δ*H*_15_ values. In these analyses, we used untresholded networks as we did in our previous studies of EEG-based functional connectivity (Racz et al., 2018, 2019, 2020; Kaposzta et al., 2021). We characterized network topology via the local (*D*) and global ($\bar{D}$) weighted node degrees from the *H*(2) and Δ*H*_15_ values of each connection, since earlier we found that in small networks, clustering coefficient and efficiency were highly correlated with node degree (Kaposzta et al., 2021). *D* represents the total connection strength of a node, while $\bar{D}$ (the average of all *D*) is an indicator of the network’s interconnectivity^2^. The calculations of *D* and $\bar{D}$ were carried out using functions of the Brain Connectivity Toolbox (Rubinov and Sporns, 2010).

### Statistical Evaluation

We evaluated between-states (e.g., Hard vs EC) and within-states (e.g., O1 vs O2 in EO) differences for both *H*(2) and Δ*H*_15_ networks. To rule out that the observed differences could be attributed to opening of the eyes, we included both resting-state periods in the statistical evaluation. Therefore, the between-states comparisons consisted of global $\bar{D}$ and local *D* comparisons of the 5 different states (EC, EO, Easy, Medium, Hard). Since the normality assumption (Lilliefors test) was not satisfied for all distributions, we used the non-parametric Friedman test. Subsequently, paired comparisons were used to identify specific pairwise differences. If any of the two populations under investigation were non-normally distributed, Wilcoxon signed-rank test was carried out. If both distributions were normal, a paired sample t-test was used. Benjamini-Hochberg (BH) correction (with α = 0.05) (Benjamini and Hochberg, 1995) was used to adjust for multiple testing. Then, we investigated the regional differences within every state’s local *D* (i.e., 91 comparisons for each of the 5 states). The same statistical tests as in the between-states comparisons were utilized. Moreover, we estimated Kendall’s coefficient of concordance (*W*) of *D* for both *H*(2) and Δ*H*_15_ networks for each state.

We also contrasted the average success rate (SR) and average reaction time (RT) between the 3 difficulty levels, applying the same statistical pipeline as described above. Then we investigated the plausible relationships between performance metrics and network architecture since scale-free FC and behavioral variables have already been shown to correlate (Ciuciu et al., 2014). In that, we examined the effect of FC on task performance by calculating the Spearman’s rank correlation (*r*) between SR-$\bar{D}$ and RT-$\bar{D}$ for each difficulty level. Every step of our analytical pipeline was implemented in MATLAB (version 2012, Mathworks, Natick, MA, United States).

## Results

### Qualitative Assessment of Bivariate Multifractal Character

Table 1 summarizes the percentage of connections passing each multifractal test. The 5 different states showed similar success rates in the spectral slope, phase randomization and Δ*H*_15_ part of shuffling tests (the latter comparing the original Δ*H*_15_ with that of shuffled surrogates). On the other hand, the rest states exhibited higher success rates in the bivariate-univariate Hurst comparison test and passed the detrended cross-correlation coefficient tests more frequently. Finally, comparing the original *H*(2) with that of shuffled surrogates had a higher success rate in the task states. As a result, more connections showed scale-free characteristics in the rest states (Table 2).

**TABLE 1:** Success rate of multifractality tests at the subject level (mean ± standard deviation).

	**Tests**					
	
	**PL**	**PR**	**SΔ*H*_15_**	**S-*H*(2)**	**DCCC**	**Biv-Univ**
**EC**	92 ± 7%	96 ± 4%	99 ± 2%	70 ± 18%	93 ± 4%	85 ± 18%
**EO**	94 ± 3%	96 ± 6%	98 ± 4%	76 ± 16%	93 ± 4%	86 ± 15%
**Easy**	93 ± 2%	97 ± 4%	99 ± 2%	90 ± 8%	64 ± 19%	65 ± 17%
**Medium**	94 ± 2%	97 ± 4%	99 ± 2%	90 ± 9%	65 ± 16%	68 ± 18%
**Hard**	94 ± 2%	97 ± 3%	99 ± 2%	89 ± 9%	62 ± 17%	73 ± 16%

*PL, power-law test; PR, phase randomization test; S-Δ*H*_15_, Δ*H*_15_ part of the shuffling test; S-*H*(2), *H*(2) part of the shuffling test; DCCC, detrended cross-correlation coefficient test; Biv-Univ, bivariate-univariate Hurst comparison.*

**TABLE 2:** Percentage of connections, at the subject level (mean ± standard deviation), that passed all multifractality assessment tests.

	**State**				
	
	**EC**	**EO**	**Easy**	**Medium**	**Hard**
***H*(2)**	48 ± 13%	55 ± 12%	31 ± 10%	34 ± 10%	35 ± 9%
**Δ*H*_15_**	46 ± 13%	53 ± 12%	30 ± 10 %	33 ± 10%	34 ± 9%

### Effect of Brain State on Multifractal Connectivity

**FIGURE 3:**
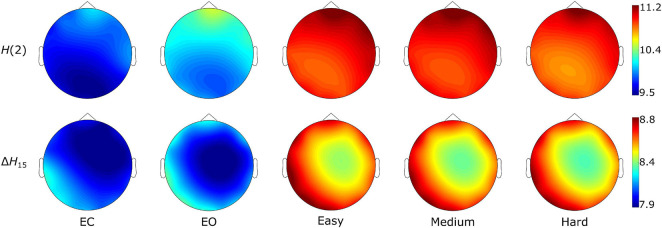
State-dependent weighted node degree topology of *H*(2) and Δ*H*_15_ brain networks. The color bars represent the values of the local node degrees.

**FIGURE 4:**
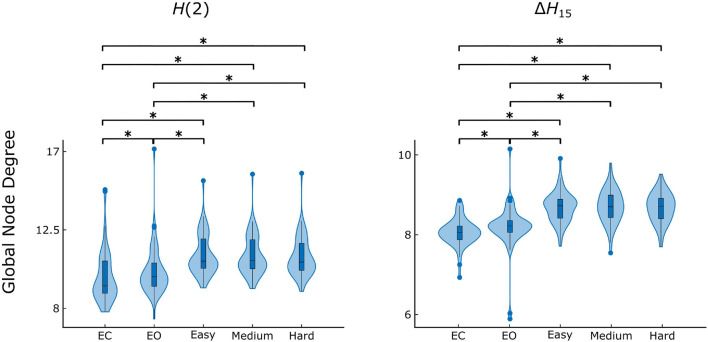
State-dependent weighted global node degree distribution of *H*(2) and Δ*H*_15_ brain networks. Significance marked by asterisk (*). Figure was created using Gramm (Morel, 2018).

**FIGURE 5:**
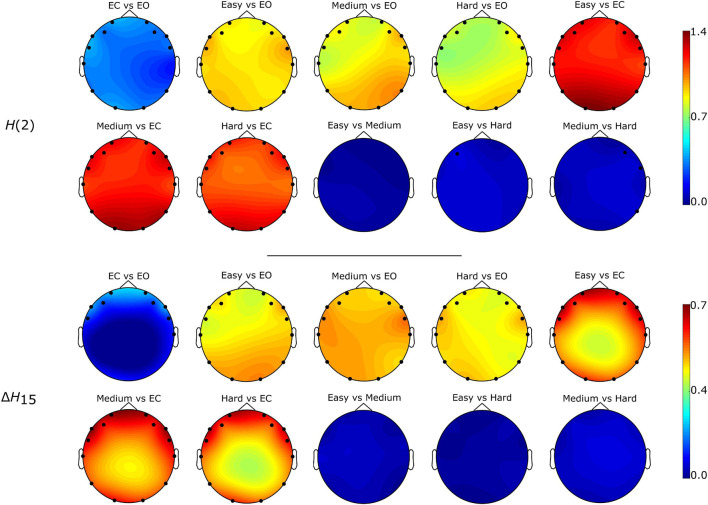
Localization of significantly different weighted node degrees for every between state comparison of the *H*(2) and Δ*H*_15_ brain networks. The colormap is based on the absolute difference of the node degrees of the states under investigation (e.g., | *D*_*O2,EC*_ - *D*_*O2,EO*_|). Only the significantly different nodes are shown.

The Friedman tests indicated a significant effect of state (*p* < 0.01), and post hoc pairwise comparisons revealed that the rest states (EC, EO) were characterized by lower $\bar{D}$ compared to the task states (Easy, Medium, Hard) (Figures 3, 4). Additionally, we found higher $\bar{D}$ during EO compared to EC, for both *H*(2) and Δ*H*_15_ networks. A similar motif emerged in the local level comparisons, with the *D* of several nodes being significantly different between the rest and task states, as well as between EC and EO for both networks (Figure 5).

**FIGURE 6:**
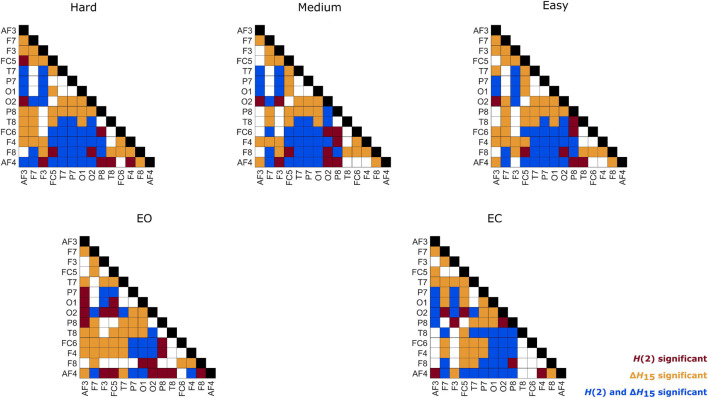
Within-state differences of node degrees in every state. Red: only *H*(2) network comparison was significant, Orange: only Δ*H*_15_ network comparison was significant, Blue: both *H*(2) and Δ*H*_15_ networks comparisons were significant.

As seen in Figure 3, the *H*(2) networks had higher FC in the frontal regions, while higher values of Δ*H*_15_ were observed in the occipital cortex. This regional variability was statistically validated by the within-state comparisons, which showed significant differences within all 5 tasks, for both *H*(2) and Δ*H*_15_ networks. We also observed that if the *D* of two nodes in the Δ*H*_15_ network were statistically different, there was a high chance of the equivalent nodes being statistically different in the *H*(2) network as well, while the opposite did not occur. This can be easily visualized by the abundance of blue [both *H*(2) and Δ*H*_15_ significant] and orange (only Δ*H*_15_ significant), in contrast to the sparse red [only *H*(2) significant] boxes in Figure 6. Moreover, small subject concordance appeared only in the Δ*H*_15_ networks; on the contrary, no subject agreement was found in the *H*(2) networks (Table 3).

**TABLE 3:** State-dependent subject concordance, as captured by Kendall’s W.

	**State**				
	
	**EC**	**EO**	**Easy**	**Medium**	**Hard**
***H*(2)**	0.10	0.09	0.09	0.12	0.11
**Δ*H*_15_**	0.24	0.15	0.25	0.24	0.26

### Cognitive Performance and Its Correlates With Functional Connectivity

**FIGURE 7:**
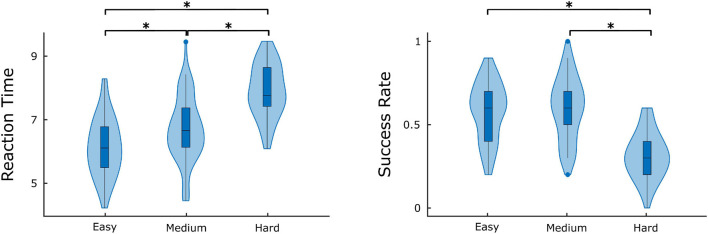
Average success rate and reaction time for different difficulty levels. Significant differences are marked by asterisk (*). Figure was created using Gramm (Morel, 2018).

**FIGURE 8:**
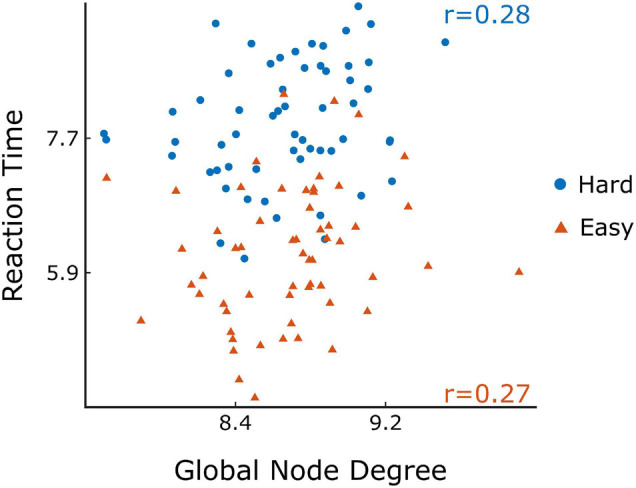
Scatter plots of the reaction time vs global node degree for Easy (orange) and Hard (blue) task in Δ*H*_15_ networks and their Spearman’s correlation (r). Figure was created using Gramm (Morel, 2018).

The comparison of difficulty levels indicated a significant decrease of SR in the Hard state. RT was also statistically different between the 3 difficulty levels, with Easy having the fastest response and Hard having the slowest (Figure 7). Furthermore, significant (*p* < 0.05) positive correlations were found between RT and $\bar{D}$ of the Δ*H*_15_ networks during Easy and Hard (Figure 8). After BH correction, these correlations were rendered not significant.

## Discussion

This study investigated the scale-free coupled dynamics of brain activity in resting state and during a visual pattern recognition task of various difficulty levels. We employed two FC estimators derived from bivariate focused-based multifractal analysis, namely *H*(2) and Δ*H*_15_. They were used for constructing brain networks based on their multifractal connectivity for both rest and task conditions. Our findings show that: (i) the local and global functional connectivity measures increased during task when compared to resting conditions, indicating a reorganization of brain networks, and (ii) there was a substantial regional variability within the 5 different states. However, significant correlations were found only between the global node degree and average reaction time during Easy and Hard tasks in the Δ*H*_15_-networks.

After acquiring the BFMF measures, *H*(2) and Δ*H*_15_, it was essential to perform an array of multifractality assessment tests since by default not all functional connections – or in general, not all dynamic processes – can be assumed to have multifractal character. Our tests showed that a considerable fraction of the connections had true multifractal characteristics (Table 1). Similar success rates have been found in the resting state previously (Stylianou et al., 2021). Despite the different channel density of the EEG devices and the different sampling populations, similar results were obtained in these studies, concluding that coupled dynamics between cortical regions are indeed multifractal during rest. The extent of multifractality decreased during task, as indicated by the lower number of connections passing our multifractality assessment tests (Table 2). To the best of our knowledge, this is the first study demonstrating the true multifractal nature of coupled dynamics during complex mental tasks. This provides an opportunity to reveal novel aspects of rest and task states using BFMF by obtaining information that would have remained hidden otherwise [for a demonstration, see the Supplementary Material in Stylianou et al. (2021)].

The higher node degree in the fully connected (i.e., unthresholded) *H*(2) and Δ*H*_15_ networks during task corresponds to increased *H*(2) and Δ*H*_15_ values of the connections. The high values of *H*(2) indicate a relative shift of the coupled dynamics toward lower frequency components. This greater long-term memory reflects a stronger coupling between the probed regions of the brain cortex. Conversely, Ciuciu and colleagues found a shift of scale-free coupled fluctuations in fMRI-BOLD signals toward the higher frequencies (i.e., decreasing Hurst-exponent), accompanied by a decrease in connectivity between resting-state networks during a motor task (Ciuciu et al., 2014). While the signs of changes were opposite, both studies showed a positive association between *H*(2) and FC change during task. This difference could possibly be attributed to the differences in imaging modality and stimulation paradigm, which should be further investigated in future studies. Moreover, based on the elevated Δ*H*_15_ values of the connections, we can conclude that the coupling between recorded EEG signals transitioned into a state with increased multifractal strength suggesting increased nonlinearity (Ashkenazy et al., 2003). Multifractal dynamics are characterized by increased dependency between different time scales. As time scale relates to frequency, one such model is formulated by assuming a relationship between the phase of lower frequencies and the amplitude of higher frequencies (He et al., 2010). In that, a stronger phase-amplitude coupling is associated with higher nonlinearity as captured by increased Δ*H*_15_ (Ashkenazy et al., 2003). Taken together, BFMF reveals that task induces a redistribution of the long-term cross-correlation in coupled dynamics as indicated by higher Hurst exponent and renders them more interdependent across different time scales as manifested by increased Δ*H*_15_. The more pronounced multifractal character of the connections can possibly be attributed to the recruitment of excitatory/inhibitory feedback loops (Poil et al., 2012) during task, whose transient is typically characterized by nonlinear dynamics (Rabinovich and Muezzinoglu, 2010). The elevated coupling [increased *H*(2)] and feedback loops (increased Δ*H*_15_) that take place in this visual pattern recognition paradigm can be ascribed to the enhanced cooperation of distant brain areas involved in various aspects of visual processing, such as recalling short-term memory and making visual comparisons.

Both *H*(2) and Δ*H*_15_ networks showed a significantly increased connectivity in task states compared to EO and EC, captured in their global and local weighted node degrees (Figures 3–5). Our results agree with the findings of a previous functional near-infrared spectroscopy study using a very similar cognitive paradigm. Racz et al. found global weighted node degree increased in the prefrontal cortex during task (Racz et al., 2017), using the scale-specific Pearson’s correlation as FC estimator. Based on these two studies, it appears that both the scale-free and scale-specific connectivity of the brain increases during visual pattern recognition. This indicates that a significant reorganization of functional brain networks takes place in response to increased mental workload. Nevertheless, definite conclusions cannot be drawn due to the different modalities (EEG vs functional near-infrared spectroscopy). It is also noteworthy that FC increased during the transition from EC to EO. Since considerable brain capacity is devoted to visual processing, opening the eyes should substantially increase brain network activity. Thus, the observed higher node degrees during EO are consistent with the manifestation of increased mental workload. It should be recalled that a shift to higher frequencies characterizes cortical desynchronization during EO, contrasting with the earlier interpretation of increased *H*(2) (i.e., shift to lower frequencies). We speculate that scale-free and oscillatory components of coupled electrophysiological activity have different origins and could be affected by the opening of the eyes differently. Previously, we have demonstrated that the global multifractal dynamics of FC are affected by the EC-EO transition (Racz et al., 2018), our present study extends these findings by revealing the local alterations in scale-free coupled dynamics (Figure 5). Still, the mental workload of EO was not as substantial as that of the pattern recognition task, since the node degrees of the EO networks differed significantly from those of the task states. On the other hand, the 3 task states (Easy, Medium and Hard) had statistically similar node degrees (Figures 3–5), even though the cognitive stimulation paradigm showed a lower success rate for more complex images (Figure 7). Similar results were found in an n-back EEG study (Kaposzta et al., 2021), in which there was no significant difference in the density, clustering coefficient and efficiency of the 2-back and 3-back brain networks. In this n-back study, the network measures decreased during task, which is in contrast with the current findings of increased FC. This apparent controversy in FC alterations between tasks has already been noticed, with n-back being the most different from the rest of the studied task conditions (Krienen et al., 2014). The use of different FC estimators could have impacted the reported results as well. Moreover, for both BFMF measures, the within-state comparisons showed apparent regional variability (Figure 6), similarly to our previous results (Stylianou et al., 2021). In that, we saw that the degree of multifractality (Δ*H*_15_) varied more than the long-term cross-correlation [*H*(2)] across the brain, in all states. Additionally, significant differences in the long-term cross-correlation were accompanied by changes in the degree of multifractality, in most cases. A possible explanation could be that multifractality results from more complex dynamics (Tel, 1988) which tend to vary more from region to region. On the other hand, this contradicts the findings of our previous resting-state study, where *H*(2) values varied the most [cf. Table 2 in Stylianou et al. (2021)]. The different electrode densities of the EEG system used in these two studies (62 vs 14 channels) could well account for the observed differences. Nonetheless, these two studies indicate that scale-free coupled dynamics do not emerge homogenously in the brain, neither in rest nor in task states, which is a motivation for further studying the multifractal properties of FC at higher spatial resolution. Furthermore, small subject concordance within the different states was observed only for the Δ*H*_15_ network (Table 3). This agrees with a previous study (Mueller et al., 2013), which found inter-subject FC variation localized mainly in the high-order association cortices in the frontal and parietal lobes, i.e., regions strongly overlapping with those we recorded EEG from.

As to the performance metrics, the Easy state was associated with faster RT than the Medium and Hard states, while significant differences in the SR were observed only between Easy-Hard and Medium-Hard (Figure 7). Even though no significant differences in the SR were observed between Easy-Medium, the RT during the Medium task was longer. We believe that a significant difference in the SR between Easy-Medium could be found by including a larger or more diverse population sample in future studies. Furthermore, no significant associations were found between the global node degrees and performance metrics (SR and RT), with the exception of positive correlations between RT and $\bar{D}$ in the Easy and Hard states of the Δ*H*_15_ networks. Similarly, in another EEG n-back study, network measures were found significantly correlated only with RT, and not with SR (Dai et al., 2017). This suggests that lower multifractality corresponds to faster pattern recognition, while the subject’s SR remains independent of scale-free coupled dynamics. These correlations did not remain significant after BH correction, suggesting that a larger, more representative sample of the population could potentially reveal significant correlations even after BH correction.

Our results derived from the main analytical pipeline are supported by further analysis accounting for the slightly different length of analyzed signals from the task states (Supplementary Material). Because the multifractal profile of a time series is influenced by its length (Grech and Pamuła, 2012; Rak and Grech, 2018), we anticipated a similar effect on our bivariate multifractal analysis (Kristoufek, 2015b); thus, we re-analyzed our dataset in a pipeline adjusted to the different lengths of analyzed pair of time series based on the different response times. The results agree with our primary analysis, indicating that the slightly varying signal length had no effect on the observed patterns. We also compared the $\bar{D}$ of every state after excluding connections that did not pass our multifractality assessment tests. While significant differences were found between rest and task states, they were of the opposite direction, i.e., $\bar{D}$ decreased during task (Supplementary Figure 1), which can be explained by the larger number of connections that passed our tests during rest (Table 2). However, there was great inconsistency among the multifractality assessment tests for every connection and task (e.g., out of the 10 Hard segments, the connection AF4-AF3 might have passed the test in only 4 of them). In order to avoid any bias, our main analysis focused on unthresholded networks. Additionally, the thresholded analysis showed significant positive correlations between $\bar{D}$-RT in the Easy and Medium states for both *H*(2) and Δ*H*_15_ networks, warranting further investigation in future studies (Supplementary Figure 2). While a growing number of publications investigates the FC-related differences between the two sexes (Zhang et al., 2018; Ýçer et al., 2020), we found no significant sex-related differences in network architecture. Since the studies mentioned above had higher spatial resolution (higher density EEG or fMRI recordings), we believe that future experiments with higher number of EEG channels might be able to reveal such differences. As to the effect of handedness, no significant differences in $\bar{D}$ were identified between the left- and right-handed participants in any state (EC, EO, Easy, Medium, Hard) or network [*H*(2) and Δ*H*_15_]. To assess the test-retest reliability, 5 of our subjects repeated the same experiment a few months later. No significant differences were found in the SR and RT between the two sessions, suggesting that our experimental paradigm can be used in further reproducibility studies. Finally, we found a moderate concordance between *H*(2) and Δ*H*_15_ values for every subject (Supplementary Table 1), indicating a relatively constant multifractal character of the connections. Further details of these analyses can be found in the Supplementary Material.

Future developments based on this study should consider the following shortcomings. Despite its sample size, the subject cohort of our study might not have been representative of the general population, thus limiting us in drawing more general conclusions. All participants were young, healthy and educated, university students or graduates. Differences observed in the multifractal FC during task could be augmented or attenuated if a larger cohort of volunteers participated. The recorded EEG signals might be affected by scalp muscle contraction (especially at the frontal and temporal sites), as shown previously (Goncharova et al., 2003). Since the spectral characteristics of electromyographic signals considerably overlap with EEG, part of the results could be attributed to activity of motor units rather than changes in local field potentials in the brain cortex. Nonetheless, independent component analysis can remove a significant part of these electromyographic contaminations (Yilmaz et al., 2019). Additionally, task-related EEG changes are not greatly affected by muscle contractions (Boytsova et al., 2016). Because during diverse tasks different brain network architectures emerge (Krienen et al., 2014), the construction of more extensive cognitive stimuli with several different paradigms should be considered. Studies found that FC changed as subjects repeated and thus learned a task (Lewis et al., 2009; Bassett et al., 2011), which warrants that our future experiments investigate the effect of learning. Additionally, the bimodality phenomenon observed in univariate focus-based multifractal analysis (Nagy et al., 2017) can be extended to the multifractal covariance scaling function with multiple scaling ranges.

As to future perspectives, it will be interesting to see the discriminatory power of multifractal FC between rest and task states at the individual level, which was beyond the scope of this study. In future studies, we intend to investigate the rest-state classification performance of BFMF compared to other measures of brain network dynamics (Racz et al., 2020). To reveal mechanistic background of scale-free coupled dynamics, further clinical trials and animal models are needed using anesthetics, antipsychotics, antiparkinsonian and other medications (Nasrallah et al., 2017). On a final note, a promising field where such visual pattern recognition task could be advantageous is in attention deficit hyperactivity disorder (ADHD) research, where brain network alterations during spatial working memory tasks have already been revealed (Jang et al., 2020).

## Conclusion

In the present study, we reconstructed brain networks from measures of scale-free coupled dynamics in resting states and during a visual pattern recognition task estimated by our novel bivariate multifractal analytical algorithm. Initially, we showed that our method could capture true multifractal coupled dynamics that varied across different brain regions. Additionally, we saw an increase in functional connectivity during the transition from rest (EC and EO) to task states, which was however, independent of task difficulty. We also found higher functional connectivity when the participants transitioned from EC to EO. These findings could well facilitate future research of scale-free functional connectivity studies with complex experimental designs in healthy and diseased populations.

## Data Availability Statement

The raw data supporting the conclusions of this article will be made available by the authors, without undue reservation.

## Ethics Statement

The studies involving human participants were reviewed and approved by Regional and Institutional Committee of Science and Research 111 Ethics of Semmelweis University (approval number: 2020/6). The patients/participants provided their written informed consent to participate in this study.

## Author Contributions

OS implemented the analytical framework, contributed to experiment design, performed experiments, data analysis and interpretation, and wrote the first draft of the manuscript. FR performed experiments and contributed to experiment design, data analysis, and manuscript development. KK, ZK, and AC performed experiments and contributed to data analysis. AY contributed to manuscript development. AE provided conceptual guidance and supervision throughout the study. PM developed the code for BFMF, specified the concept and aims of the study and contributed to experiment design. All authors contributed to reviewing the manuscript and approved its final version.

## Conflict of Interest

The authors declare that the research was conducted in the absence of any commercial or financial relationships that could be construed as a potential conflict of interest.

## Publisher’s Note

All claims expressed in this article are solely those of the authors and do not necessarily represent those of their affiliated organizations, or those of the publisher, the editors and the reviewers. Any product that may be evaluated in this article, or claim that may be made by its manufacturer, is not guaranteed or endorsed by the publisher.

## Footnotes

1 18 recordings were carried out at 256 Hz sampling rate which were downsampled to 128 Hz prior to further preprocessing.

2 *D* $\sum_{i=1}^{n}c_{i}$where *n* represents all possible edges of a node, while *c*_*i*_ is the strength [either *H*(2) or Δ*H*_15_] of the i^th^ connection.$\bar{D}=\frac{\sum_{j=1}^{N}D_{j}}{N}$ where *N* represents all nodes of the network, while *D_*j*_^*W*^* is the weighted degree of the j^th^ node.
