# Supplementary material for: Multifractal Functional Connectivity Analysis of Electroencephalogram Reveals Reorganization of Brain Networks in a Visual Pattern Recognition Paradigm
Source: Front Hum Neurosci. 2021 Oct 18;15:740225. doi: 10.3389/fnhum.2021.740225 (PMC8558231; doi:10.3389/fnhum.2021.740225)
Supplement: Supplementary file 1 [file Data_Sheet_1.PDF]

Supplementary Material

1 Length-Adjusted Analysis

Since the multifractal profile of a time series is influenced by the number of datapoints in the time series (Grech & Pamuła, 2012; Rak & Grech, 2018), we anticipated a similar effect in our bivariate multifractal analysis (Kristoufek, 2015). Therefore, it was necessary to reanalyze our dataset in a length-adjusted pipeline to see if the observed rest-task differences could be attributed to the different lengths of the respective signals. Each subject had 30 trial (10 Easy, 10 Medium, 10 Hard) segments. The average reaction time was calculated for each difficulty level. Then, one segment of different lengths from the EC and EO was selected based on the average reaction time associated with the given difficulty level. For example, if the average reaction time for Hard was 9 seconds and the average reaction time for Easy was 6 seconds, we selected two different EO segments. Thus, the segment corresponding to Hard (EO_Hard) was 9 (average reaction time) + 10 (inter-task interval) = 19 seconds long, while the segment corresponding to Easy (EO Easy) was 6 + 10 = 16 seconds long. The starting point of each new segment was randomly selected to be between the first 30 and 120 seconds. Subsequently, we compared the global node degrees between length-adjusted rest and task states. Accordingly, 6 different comparisons (EC_Easy vs Easy, EC_Medium vs Medium, EC_Hard vs Hard, EO Easy vs Easy, EO Medium vs Medium, EO Hard vs Hard) were made. The same paired comparisons and Benjamini-Hochberg (BH) correction (with α=0.05) (Benjamini & Hochberg, 1995) as in the main analysis were carried out. The task states had higher global node degrees than the rest states for both $H(2)$ and ΔH_{15} networks, except for the EO-Easy comparison, which was not statistically significant in the case of the ΔH_{15} networks.

2 Thresholded Results

Each connectivity matrix was thresholded in order to exclude spurious connections. The ΔH_{15} network consisted of connections that passed all the multifractality tests. On the other hand, the $H(2)$ network included connections with spurious multifractality (connections that failed the ΔH_{15} part of shuffling test but passed its $H(2)$ counterpart were included), as long as they passed the rest of the tests (spectral slope, detrended cross-correlation coefficient and bivariate-univariate Hurst comparison); since a connection can express long-term cross-correlation [captured by $H(2)$] without having a true multifractal character (captured by ΔH_{15}). Then, using the same pipeline as in the main manuscript, we compared the: *i*) global node degree between the 5 different states and *ii*) relationship between global node degree and performance metrics. As seen in **Figure S1** additional to the differences observed in the main analysis, we also see significant differences between Easy-Medium and Easy-Hard. Even though the main analysis showed increased FC during task, the comparisons of thresholded networks found the opposite pattern (i.e., FC decreased during task). Finally, we observed positive Spearman's correlations between the global node degree and reaction time for the Easy and Medium tasks in both $H(2)$ and ΔH_{15} networks (**Figure S2**), even after BH correction.

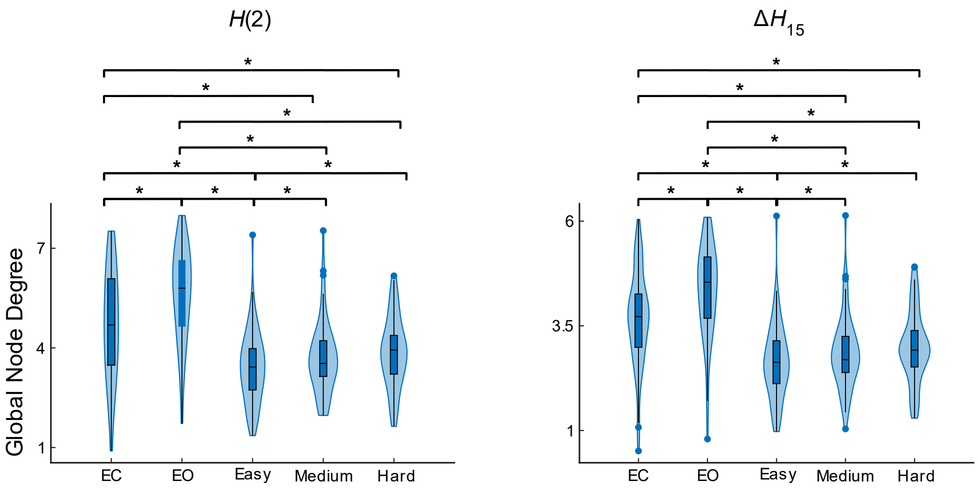

Figure S1 | *State-dependent weighted global node degree distribution of H(2) and ΔH₁₅ brain networks*. Significance marked by *. Figure was created using Gramm (Morel, 2018).

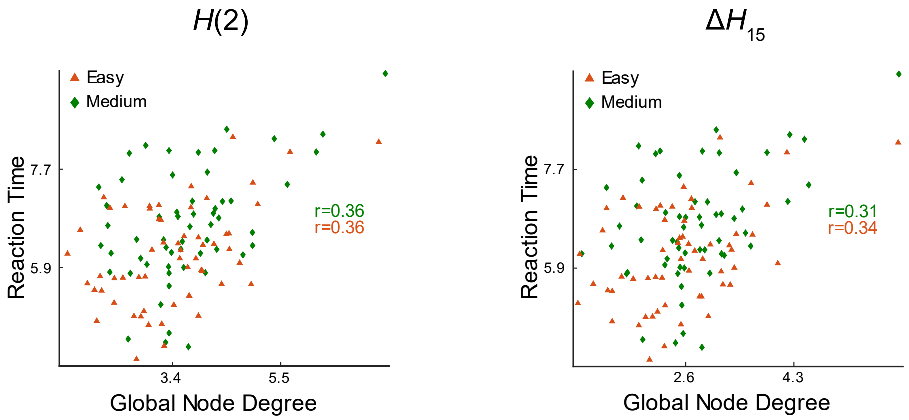

Figure S2 | *Scatter plots of the reaction time vs global node degree for Easy (orange) and Medium (green) task and their Spearman's correlation (r)*. Panel A: $H(2)$ networks. Panel B: ΔH_{15} networks. Figure was created using Gramm (Morel, 2018).

3 Sex and Handedness Differences

Recently, both EEG and fMRI FC studies have shown significant differences between the two sexes (İçer, Acer, & Baş, 2020; C. Zhang, Dougherty, Baum, White, & Michael, 2018). In order to explore the effect of gender, we compared the global node degrees for both $H(2)$ and ΔH_{15} networks in all 5 states [i.e. $H(2)$ Hard, $H(2)$ Medium, ΔH_{15} EO etc.]. Paired comparisons were used to identify specific differences. If any of the two distributions under investigation was non-normal, Wilcoxon rank-sum test was carried out. If both distributions were normal, a two-sample t-test was used (after taking into consideration the relationship between the variances of the two distributions). BH correction (with α=0.05) was used to adjust for multiple testing. The same analysis was performed once again, but instead of dividing the subjects into male and female groups, we separated them according to

handedness. No significant differences were found neither between the two sexes nor between left- and right-handed individuals.

4 Reproducibility

To investigate the reproducibility of our experimental paradigm and FC estimation methods, we compared the behavioral metrics and concordance of $H(2)$ and ΔH_{15} in 5 of our participants who repeated the task a few months later. The similarity of success rate and reaction time in the two occasions was investigated by a series of paired comparisons for every difficulty level. If any of the two distributions under investigation was non-normal, Wilcoxon signed-rank test was carried out. If both distributions were normal, a paired sample t-test was used. BH correction (with α=0.05) was used after acquiring all p values. The concordance for all $H(2)$ and ΔH_{15} networks was also calculated. This was done by calculating Kendall's W in the two different occasions for every subject (91x2 matrix for each state and subject). No significant difference was found in the success rates and reaction times between the two occasions in any of the task states (Easy, Medium and Hard), while moderate to strong subject concordance was found in every state for both $H(2)$ and ΔH_{15} networks (**Table S1**).

Table S1 | *Subject concordance in the two occasions, as captured by Kendall's W (mean±standard deviation).*

**BFMF-Output**	**State**				
	**EC**	**EO**	**Easy**	**Medium**	**Hard**
$\boldsymbol{H(2)}$	0.56±0.13	0.56±0.08	0.56±0.10	0.52±0.05	0.53±0.08
$\boldsymbol{\Delta H_{15}}$	0.64±0.09	0.64±0.12	0.57±0.14	0.63±0.12	0.63±0.15

5 References

Benjamini, Y., & Hochberg, Y. (1995). Controlling the False Discovery Rate: A Practical and Powerful Approach to Multiple Testing. *Journal of the Royal Statistical Society: Series B (Methodological)*, *57*(1), 289–300. https://doi.org/10.1111/j.2517-6161.1995.tb02031.x

Grech, D., & Pamuła, G. (2012). Multifractal Background Noise of Monofractal Signals. *Acta Physica Polonica A*, *121*(2B), B-34-B-39. https://doi.org/10.12693/APhysPolA.121.B-34

İçer, S., Acer, İ., & Baş, A. (2020). Gender-based functional connectivity differences in brain networks in childhood. *Computer Methods and Programs in Biomedicine*, *192*, 105444. https://doi.org/10.1016/j.cmpb.2020.105444

Kristoufek, L. (2015). Finite sample properties of power-law cross-correlations estimators. *Physica A: Statistical Mechanics and Its Applications*, *419*, 513–525. https://doi.org/10.1016/j.physa.2014.10.068

Morel, P. (2018). Gramm: grammar of graphics plotting in Matlab. *The Journal of Open Source Software*, *3*(23), 568. https://doi.org/10.21105/joss.00568

Rak, R., & Grech, D. (2018). Quantitative approach to multifractality induced by correlations and broad distribution of data. *Physica A: Statistical Mechanics and Its Applications*, *508*, 48–66. https://doi.org/10.1016/j.physa.2018.05.059

Zhang, C., Dougherty, C. C., Baum, S. A., White, T., & Michael, A. M. (2018). Functional connectivity predicts gender: Evidence for gender differences in resting brain connectivity. *Human Brain Mapping*, *39*(4), 1765–1776. https://doi.org/10.1002/hbm.23950

Zhang, J., Dong, X., Wang, L., Zhao, L., Weng, Z., Zhang, T., … Yan, T. (2018). Gender Differences in Global Functional Connectivity During Facial Emotion Processing: A Visual MMN Study. *Frontiers in Behavioral Neuroscience*, *12*. https://doi.org/10.3389/fnbeh.2018.00220
